# Septic embolism of the lung due to spondylodiscitis

**DOI:** 10.1590/0037-8682-0662-2021

**Published:** 2022-04-08

**Authors:** Fatih Hakan Tufanoğlu, Behiç Akyüz, Süleyman Bekirçavuşoğlu

**Affiliations:** 1 Bursa City Hospital, Department of Radiology, Bursa, Turkey.

**FIGURE 1:**
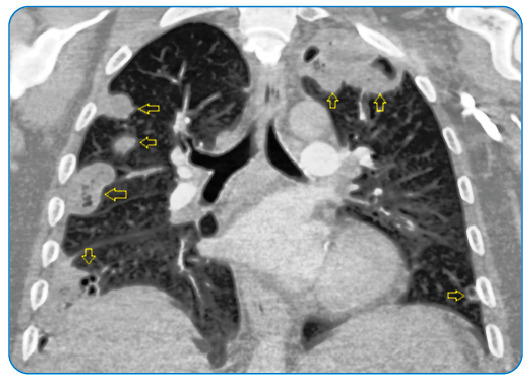
Coronal reformatted chest CT shows peripherally distributed nodules with or without cavitation (open yellow arrows).

**FIGURE 2:**
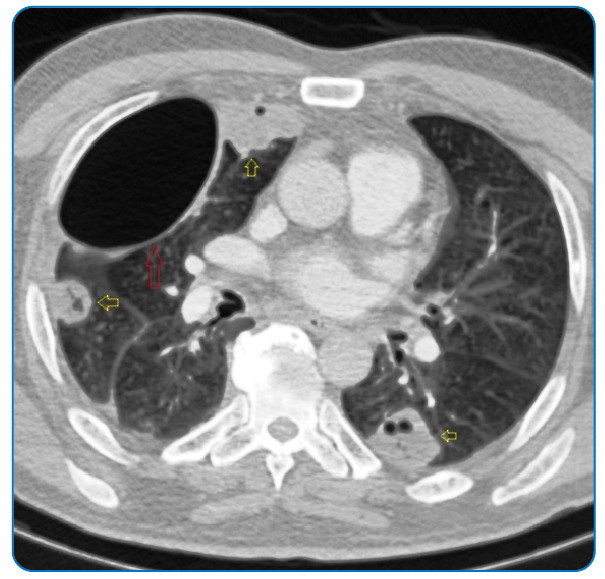
Axial chest CT shows cavitary subpleural nodules (open yellow arrows) and residual pneumothorax (open red arrow).

**FIGURE 3:**
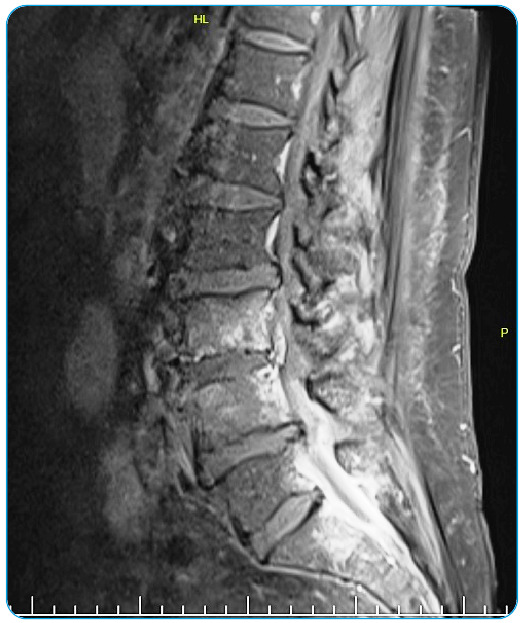
Contrast-enhanced T1-weighted fat saturated magnetic resonance imaging shows L3-4 and S1 spondylodiscitis with epidural enhancement.

A 57-year-old male with respiratory distress and back and chest pain was admitted to the respiratory disease clinic in September 2021. He was referred from another hospital for right-sided spontaneous pneumothorax on radiographic examination. On arrival, he was tachypneic, and laboratory studies showed leukocytosis (32 × 10^3^ µL) and an elevated level of C-reactive protein (214 mg/L). Computed tomography (CT) of the thorax revealed right-sided pneumothorax and bilateral peripherally distributed multiple nodules with cavitation (Figures 1-2). Antibiotics were started and a chest tube was inserted. Blood cultures were negative, but the bronchial lavage culture revealed *Staphylococcus aureus*. The biopsy of the lung nodules showed lymphoplasmacytic infiltration and inflammation. A week after admission, the patient complained of leg numbness. Lumbar magnetic resonance imaging revealed spondylodiscitis of L3-4 and S1 (Figure 3). After 6 weeks of treatment with levofloxacin, the pulmonary lesions regressed. The patient refused surgery for spondylodiscitis.

Septic pulmonary embolization is a rare condition that is difficult to diagnose due to nonspecific clinical and radiological findings. Indwelling catheters, drug abuse, and infective endocarditis are risk factors for this condition^1^. The CT appearance of septic emboli includes well-defined peripherally located nodules with or without cavitation or wedge-shaped peripheral lesions^2^. Feeding vessel signs were also observed.

Patients rarely present with spontaneous pneumothorax^3^. In patients with spondylodiscitis and peripherally distributed cavitary nodules on CT scan, septic lung emboli should be suspected.
